# Immunoreactivity to WT1 peptide vaccine is associated with prognosis in elderly patients with acute myeloid leukemia: follow-up study of randomized phase II trial of OCV-501, an HLA class II-binding WT1 polypeptide

**DOI:** 10.1007/s00262-023-03432-4

**Published:** 2023-04-24

**Authors:** Tomoki Naoe, Akiko Saito, Nahoko Hosono, Senji Kasahara, Hideharu Muto, Kaoru Hatano, Mizuki Ogura, Taro Masunari, Masatsugu Tanaka, Kensuke Usuki, Yuichi Ishikawa, Koji Ando, Yukio Kondo, Yusuke Takagi, Satoru Takada, Maho Ishikawa, Ilseung Choi, Akihiro Sano, Hirokazu Nagai

**Affiliations:** 1 National Hospital Organization Nagoya Medical Center, 4-1-1 San-No-Maru, Naka-Ku, Nagoya, Japan; 2 Clinical Research Center, National Hospital Organization Nagoya Medical Center, Nagoya, Japan; 3 Clinical Research Center, National Hospital Organization Headquarters, Tokyo, Japan; 4 Department of Hematology and Oncology, University of Fukui, Fukui, Japan; 5 Department of Hematology, Gifu Municipal Hospital, Gifu, Japan; 6 Department of Blood Transfusion, Tokyo Metropolitan Otsuka Hospital, Tokyo, Japan; 7 Department of Hematology, Jichi Medical University Hospital, Shimotsuke, Japan; 8 Department of Hematology, Japanese Red Cross Medical Center, Tokyo, Japan; 9 Department of Hematology/Infectious Diseases, Chugoku Central Hospital, Fukuyama, Japan; 10 Department of Hematology, Kanagawa Cancer Center, Yokohama, Japan; 11 Department of Hematology, NTT Medical Center Tokyo, Tokyo, Japan; 12 Department Hematology and Oncology, Nagoya University Graduate School of Medicine, Nagoya, Japan; 13 Department of Hematology, Nagasaki University Hospital, Nagasaki, Japan; 14 Department of Hematology, Toyama Prefectural Central Hospital, Toyama, Japan; 15 Department of Hematology, Ogaki Municipal Hospital, Ogaki, Japan; 16 Leukemia Research Center, Saiseikai Maebashi Hospital, Maebashi, Japan; 17 Department of Hemato-Oncology, Saitama Medical University International Medical Center, Hidaka, Japan; 18 Department of Hematology and Cell Therapy, National Hospital Organization Kyushu Cancer Center, Fukuoka, Japan; 19 Department of Hematology, National Hospital Organization Nagoya Medical Center, Nagoya, Japan

**Keywords:** Acute myeloid leukemia (AML), Immunotherapy, Peptide vaccine, WT1, Biomarker

## Abstract

We previously conducted a randomized phase II trial of OCV-501, a WT1 peptide presented by helper T cells, in elderly AML (acute myeloid leukemia) patients in first remission, indicating no difference in 2-year disease-free survival (DSF) between the OCV-501 and placebo groups. Here, we analyzed 5-year outcome and biomarkers. Five-year DFS was 36.0% in the OCV-501 group (N = 52) and 33.7% in the placebo group (N = 53), with no significant difference (*p* = 0.74). The peripheral *WT1* mRNA levels were marginally suppressed in the OCV-501 group compared with the placebo group. Enhanced anti-OCV-501 IgG response by the 25th week was an independent favorable prognostic factor. Anti-OCV-501 IFNγ responses were less frequent than the IgG reactions. These findings suggest that host immunoreactivity has a significant impact on the prognosis of AML and that further improvement of the WT1 peptide vaccine is needed.

**Supplementary Information:**

The online version contains supplementary material available at 10.1007/s00262-023-03432-4.

## Introduction

The prognosis of elderly patients with acute myeloid leukemia (AML) is poor because of leukemia characteristics, patients’ comorbidities, and treatment toxicities [1, 2]. Even if remission is achieved by chemotherapy, post-remission therapy has not been standardized [3, 4]. Molecular target therapies for AML have been developed, but a problem of resistance emerges [5]. Thus, there is a need for immunotherapy that causes less physical stress and no-cross resistance to the above therapies.

The *WT1* gene is highly expressed in hematopoietic and various solid tumors, and cancer immunotherapies targeting WT1 protein have been developed [6–8]. Regarding WT1 peptide vaccines, however, there are few reports of studies that have verified its clinical usefulness based on randomized trials. Furthermore, biomarkers related to WT1 vaccine therapy remain unclear.

OCV-501, a tumor vaccine, is an HLA class II-binding polypeptide consisting of 16 amino acid residues derived from WT1 protein [9, 10]. Previous studies revealed that OCV-501 induces not only peptide-specific Th1 cells but also WT1-specific cytotoxic T-lymphocytes, suggesting its potential as a cancer vaccine [9]. From 2013 to 2017, a "multicenter, randomized, placebo-controlled, double-blind, comparative study (Phase II) to evaluate the efficacy and safety of OCV-501 in elderly patients with AML" (referred to as “OCV-501 Phase II trial”) was conducted [11]. Patients with AML aged 60 years or older who had achieved their first complete remission (CR) were randomized to receive OCV-501 (N = 69) or placebo (N = 65), administered the vaccine, and observed for 2 years. Median DFS was 12.1 and 8.4 months in the OCV-501 and placebo groups, respectively, without a significant difference. However, elevated antibody titers or specific T-cell immune responses to OCV-501 were associated with a favorable prognosis. Furthermore, the 2-year DFS rate was approximately 40% in both groups, being higher than expected on planning the OCV-501 Phase II trial. Here we followed the efficacy additionally for 4.2 years (median) and analyzed peripheral *WT1* mRNA levels and WT-1-specific immunoreactivity from the perspective of whether they are predictive biomarkers for vaccine efficacy and prognosis (UMIN-CTR ID: UMIN000045499).

## Materials and methods

### Study design and patients

Among 134 patients who were randomized in the multicenter, randomized, double-blind, placebo-controlled phase II study (ClinicalTrials.gov: NCT01961882), one patient allocated to the OCV-501 group did not receive the study drug, and 28 patients enrolled from overseas institutions were excluded from this study. Of the 105 eligible patients, 52 were the OCV-501 group and 53 were the placebo group. At the end of the OCV-501 phase II trial, 58 of the 105 patients were alive and 47 had died. Among the 58 surviving patients, updated information was obtained from 52 patients and censored data at the end of the trial were used in the remaining 6 patients. The study procedure and assessment were described previously [11].

Briefly, AML patients who were ≥ 60 years, achieved initial CR with one or two cycles of induction chemotherapy, finished consolidation, and were not eligible for transplantation were enrolled. Since binding of OCV-501 to HLA class II molecules can be expected in 83.8–98.8% of Japanese [10], HLA genotyping was not performed for patient recruitment. Patients were randomly assigned to receive either OCV-501 emulsified with Montanide ISA 51 adjuvant (Seppic Inc., Pris, France) or placebo (adjuvant only) for 2 years. Blood was collected to evaluate the *WT1* mRNA expression level and anti-OCV-501 antibody at least once every four weeks until 2 years or relapse. Peripheral blood was collected in the 1st, 5th, 9th, and 13th weeks, and every 12 weeks after the 13th week to assess OCV-501-specific interferon γ (IFNγ) production. The primary endpoint was 5-year disease-free survival (DFS), and secondary endpoints were 5-year overall survival (OS) and interactions between immunoreactivities to OCV-501 and the prognosis and between peripheral *WT1* mRNA levels and the prognosis.

### Statistical analysis

DFS was defined as the length of time from the date of vaccination to any recurrent disease or death, whichever occurred first. OS was defined as the length of time from the date of vaccination to death from any cause. Survival curves were estimated using the Kaplan–Meier method and compared using log-rank tests. Univariate and multivariate analyses were performed using the Cox proportional hazards regression model. The Wilcoxon rank-sum test, Fisher’s exact test, and chi-square test were used to investigate the relationships among various clinical and biological parameters. *p*-values less than 0.05 were considered significant. Statistical analyses were performed using the statistical software R: A language and environment for statistical computing (ver 4.1.2, R Core Team (2021), R Foundation for Statistical Computing, Vienna, Austria).

## Results

**Fig. 1:**
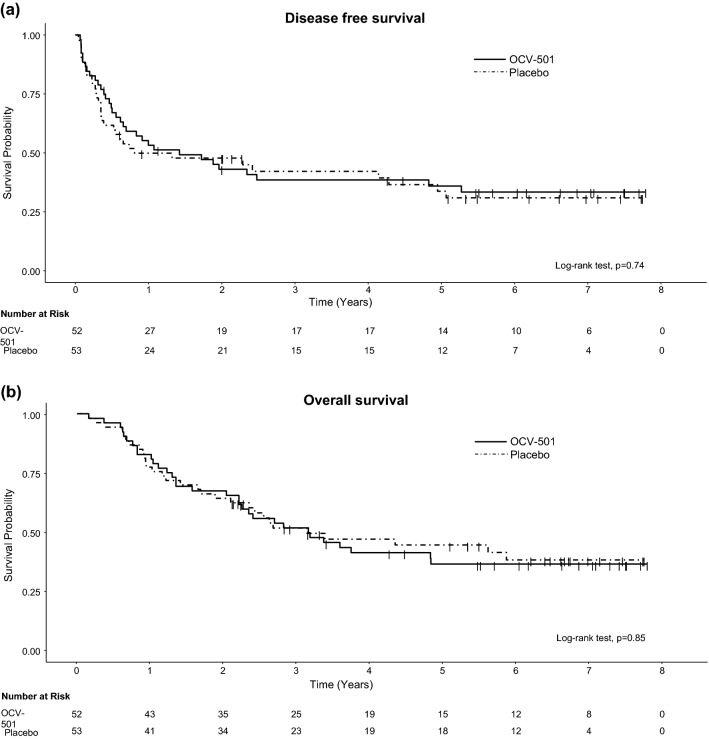
DFS **a** and OS **b** in the OCV-501 and placebo groups

The patients’ characteristics are presented in Supplementary Table 1. The 5-year DFS rate (95% confidence interval, 95%CI) was 36.0 (22.8–49.3)% in the OCV-501 group and 33.7 (20.2–47.8)% in the placebo group, indicating no significant difference (*p* = 0.74, by the log-rank test, Fig. 1). Relapse was reported in 32 of 52 (61.5%) patients in the OCV-501 group and 32 of 53 (60.4%) patients in the placebo group. The 5-year OS rates (95% CI) in the OCV-501 and placebo groups were 36.3 (23.0–49.6)% and 44.4 (30.1–57.7)%, respectively (*p* = 0.85 by the log-rank test).

We analyzed the effect of vaccination on peripheral *WT1* mRNA levels that were measured every 4 weeks for 2 years in peripheral blood (Supplementary Fig. 1). The median *WT1* mRNA level before vaccination was 50.0 copies/cg (49.0–12,000). The individual peak level of *WT1* mRNA during the vaccination was lower in the OCV-501 group than in the placebo group, although the difference was marginal (295.0 vs. 910.0/µg RNA, respectively, *p* = 0.078 by Wilcoxon rank sum test, Table 1).

**Table 1:** (a**)** Peripheral ***WT1* mRNA peak levels (copy/**µ**g RNA) in the OCV-501 and placebo groups. **(**b) *WT1* mRNA category and distribution**

(a)	OCV501	Placebo
Number of patients	52	53
Mean (SD)	4,988.6 (11,255.0)	6,436.0 (15,129.2)
Median	295.0	910.0
Range	49.0–54,000.0	49.0–77,000.0

(b)	OCV501	Placebo
Low (min ~ ≦ Q1)	18 (34.6%)	9 (17.0%)
Intermediate (Q1 < ~ ≦ Q3)	22 (42.3%)	30 (56.6%)
High (Q3 < ~ max)	12 (23.1%)	14 (26.4%)
Min = 49.0, Q1 = 120.0, Q3 = 3800.0 copy/µg RNA		

**Fig. 2:**
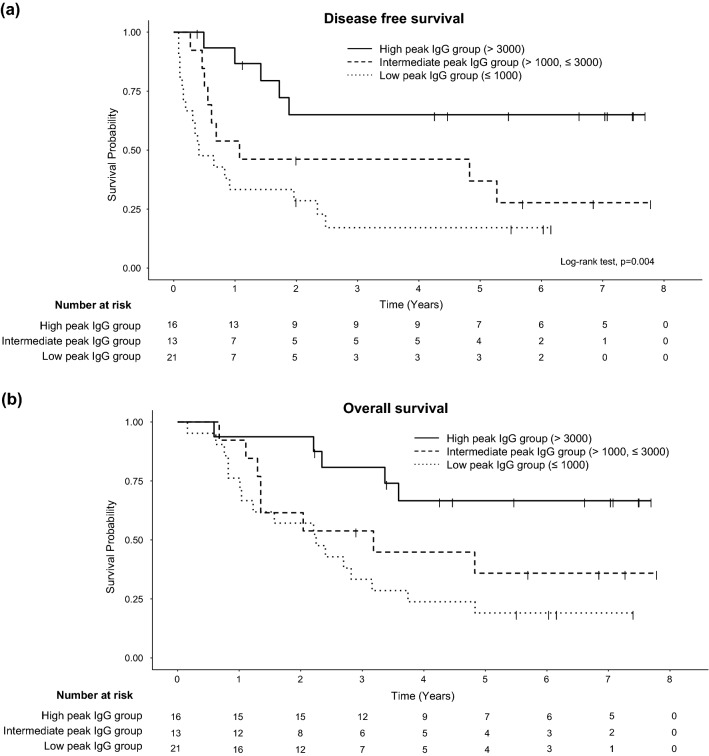
The peak anti-OCV-501 IgG levels until the 25th week are correlated with prognosis. DFS (**a**) and OS (**b**). The OCV-501 group was stratified by the peak anti-OCV-501 IgG levels (low ≤ 1000 ng/mL, 1000 < middle ≤ 3000 ng/mL, 3000 ng/mL < high)

Next, we analyzed the association between DFS and WT1-specific immunoreactivity. Enhanced WT1-specific IgG responses were observed in the OCV-501 group by the 25th week (Suppl. Figure 2) and this event was not related to age, sex, ECOG score, or myelodysplastic syndromes (MDS)-related changes (data not shown). Peak anti-OCV-501 IgG levels by the 25th week were correlated with the prognosis (*p* = 0.004, by log-rank test, Fig. 2) The IgG response was an independent prognostic factor (Supplementary Table 2).

**Fig. 3:**
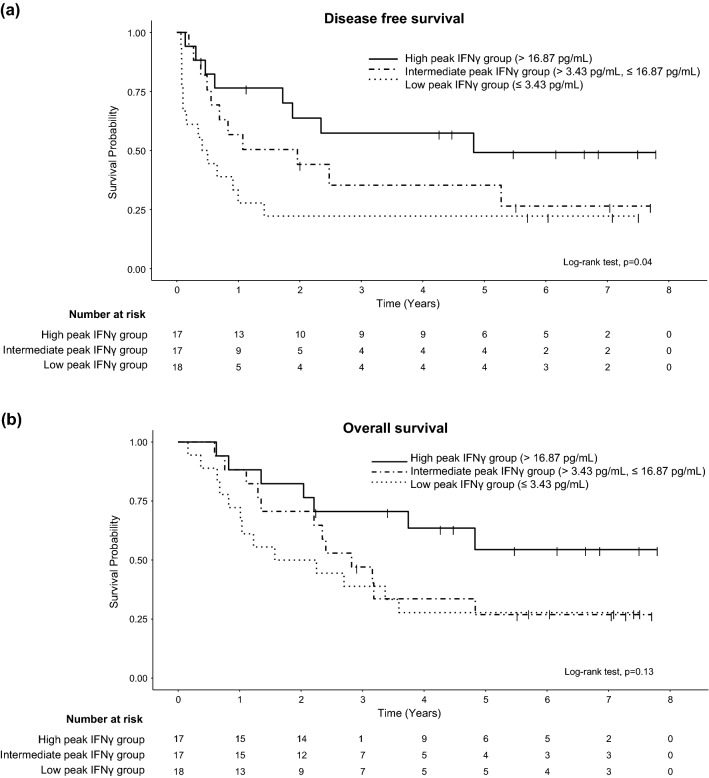
The peak OCV-501-specific IFNγ level correlates with prognosis. DFS (**a**) and OS (**b**). All patients were stratified by the peak IFNγ levels (low ≤ Q1 = 0.8 pg/mL, Q1 < middle ≤ Q3 = 18.5 pg/mL, Q3 < high)

Although post-vaccination anti-OCV-501 IFNγ peak levels were also associated with DFS and OS (Fig. 3), the IFNγ response to OCV-501 was less frequent and less persistent than the IgG response (Supplementary Fig. 3).

## Discussion

This study revealed that 1) there was no prognostic difference between the OCV-501 and placebo groups even on long-term follow-up, 2) *WT1* mRNA levels were marginally suppressed in the OCV-501 group compared with placebo group, 3) the anti-OCV-501 IgG response until the 25th week was independently associated with favorable survival, 4) the anti-OCV-501 IFNγ response was unstable but associated with the prognosis.

Although the first finding is disappointing, the modest suppression of peripheral *WT1* mRNA expression levels was encouraging for the WT1 vaccine development. When *WT1* mRNA expression was at a low level, its suppressive effect was obvious (Supplementary Fig. 1, Table 1b), and a correlation was observed between the IgG increase and the suppression of *WT1* mRNA expression (data not shown). It is expected that this phenomenon reflects the efficacy of vaccines targeting minimal residual disease [12]. Improvement of the vaccination, including dosing methods, intervals, and combination [13], will be necessary, as will the development of non-peptide immunization, as discussed below.

The third finding regarding IgG reactivity is a characteristic observed with this helper peptide. The most likely reason why OCV-501 did not contribute to improved prognosis overall despite IgG reactivity being correlated with prognosis is that vaccine responsiveness reflects general immune functions in AML patients, and that those who retain the functions may have a better prognosis irrespective the vaccine effect.

Recently, there have been reports that there is a difference in immune functions in the bone marrow between a group of patients showing long-term remission with chemotherapy alone and a group of patients showing relapse [14]. Immune-related gene expressions are also related to survival in AML patients [15, 16].

Regarding the fourth finding, the IFNγ-response was observed not only in the OCV-501 group but also in a small number of the placebo group, and both the IFNγ-response subgroups showed a trend towards a better prognosis than the IFNγ-nonresponse subgroup (data not shown). Cell-mediated immunity may have been enhanced by the adjuvants, which might be associated with that 5-year DFS rates exceeded 30% in both groups. WT1-specific cytotoxic T-cells were reportedly detected in AML patients with longer survival [17].

This study shows that responsiveness to the WT1 vaccine in AML patients in CR is variable. For immune-response groups, it will be necessary to improve vaccines to induce earlier responses. For immunocompromised groups, passive immunotherapy, targeting immune suppression or a new kind of WT1 vaccine should be developed [18]. Since immunoreactivity does not correlate with AML classification or age, predicting such reactivity presents a new challenge. Analysis of lymphocyte parameters with or without WT1 peptide specificity is extremely important from the point of view of clinical utility.

### Supplementary Information

Below is the link to the electronic supplementary material.

Supplementary file1 (PPTX 252 KB)

Supplementary file2 (DOCX 15 KB)

## Data Availability

The datasets generated during and/or analyzed during the current study are available from the corresponding author on reasonable request.
