# Supplementary material for: Immunoreactivity to WT1 peptide vaccine is associated with prognosis in elderly patients with acute myeloid leukemia: follow-up study of randomized phase II trial of OCV-501, an HLA class II-binding WT1 polypeptide
Source: Cancer Immunol Immunother. 2023 Apr 24;72(8):2865–71. doi: 10.1007/s00262-023-03432-4 (PMC10123586; doi:10.1007/s00262-023-03432-4)
Supplement: Supplementary file 1 — Supplementary file1 (PPTX 252 KB) [file 262_2023_3432_MOESM1_ESM.pptx]

## Slide 1
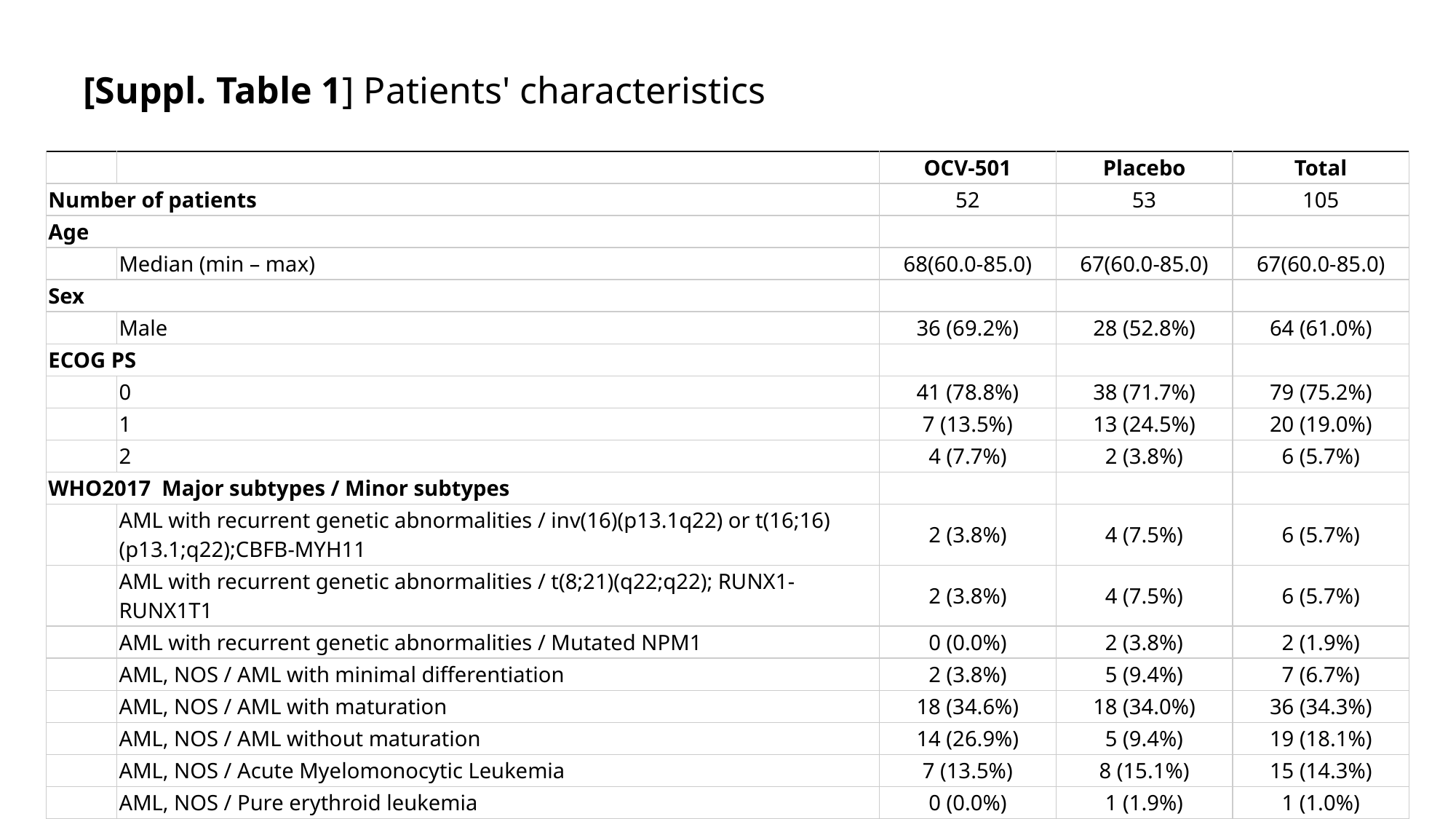

# [Suppl. Table 1] Patients' characteristics
| | | OCV-501 | Placebo | Total |
| --- | --- | --- | --- | --- |
| Number of patients | | 52 | 53 | 105 |
| Age | | | | |
| | Median (min – max) | 68(60.0-85.0) | 67(60.0-85.0) | 67(60.0-85.0) |
| Sex | | | | |
| | Male | 36 (69.2%) | 28 (52.8%) | 64 (61.0%) |
| ECOG PS | | | | |
| | 0 | 41 (78.8%) | 38 (71.7%) | 79 (75.2%) |
| | 1 | 7 (13.5%) | 13 (24.5%) | 20 (19.0%) |
| | 2 | 4 (7.7%) | 2 (3.8%) | 6 (5.7%) |
| WHO2017 Major subtypes / Minor subtypes | | | | |
| | AML with recurrent genetic abnormalities / inv(16)(p13.1q22) or t(16;16)(p13.1;q22);CBFB-MYH11 | 2 (3.8%) | 4 (7.5%) | 6 (5.7%) |
| | AML with recurrent genetic abnormalities / t(8;21)(q22;q22); RUNX1-RUNX1T1 | 2 (3.8%) | 4 (7.5%) | 6 (5.7%) |
| | AML with recurrent genetic abnormalities / Mutated NPM1 | 0 (0.0%) | 2 (3.8%) | 2 (1.9%) |
| | AML, NOS / AML with minimal differentiation | 2 (3.8%) | 5 (9.4%) | 7 (6.7%) |
| | AML, NOS / AML with maturation | 18 (34.6%) | 18 (34.0%) | 36 (34.3%) |
| | AML, NOS / AML without maturation | 14 (26.9%) | 5 (9.4%) | 19 (18.1%) |
| | AML, NOS / Acute Myelomonocytic Leukemia | 7 (13.5%) | 8 (15.1%) | 15 (14.3%) |
| | AML, NOS / Pure erythroid leukemia | 0 (0.0%) | 1 (1.9%) | 1 (1.0%) |
| | AML with myelodysplasia-related changes | 7 (13.5%) | 6 (11.3%) | 13 (12.4%) |

## Slide 2
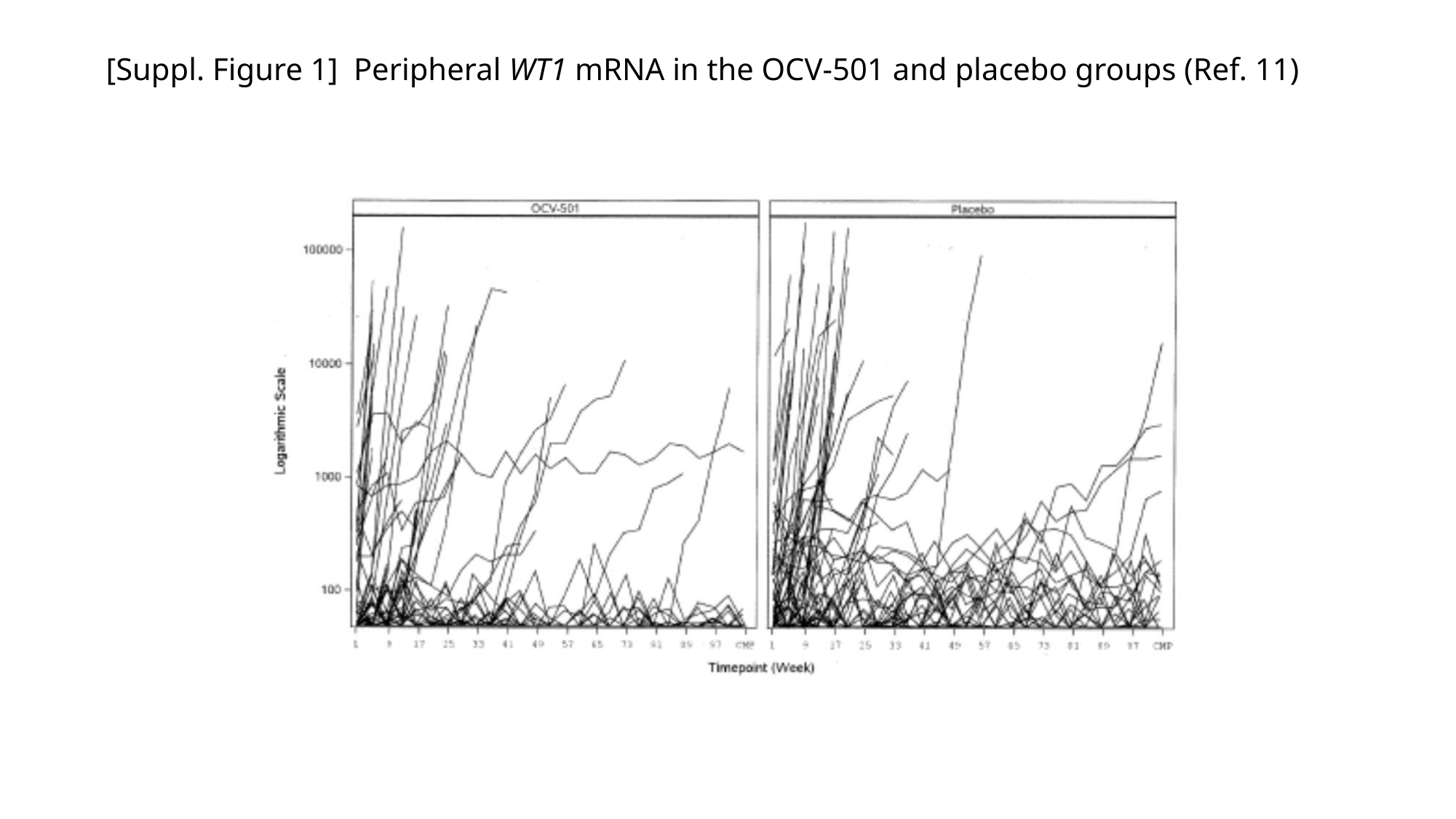

[Suppl. Figure 1] Peripheral WT1 mRNA in the OCV-501 and placebo groups (Ref. 11)

## Slide 3
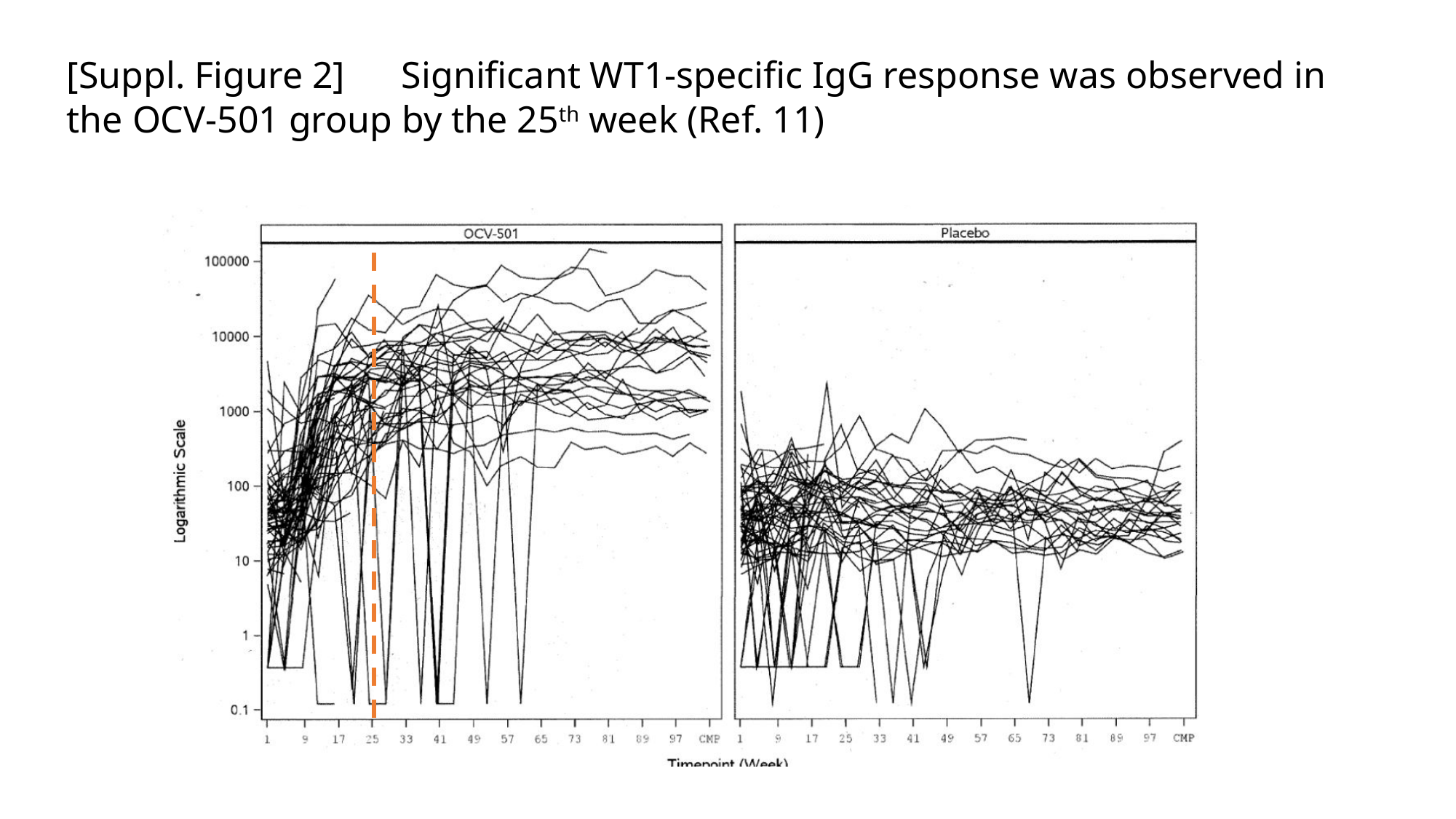

[Suppl. Figure 2]　Significant WT1-specific IgG response was observed in the OCV-501 group by the 25th week (Ref. 11)

## Slide 4
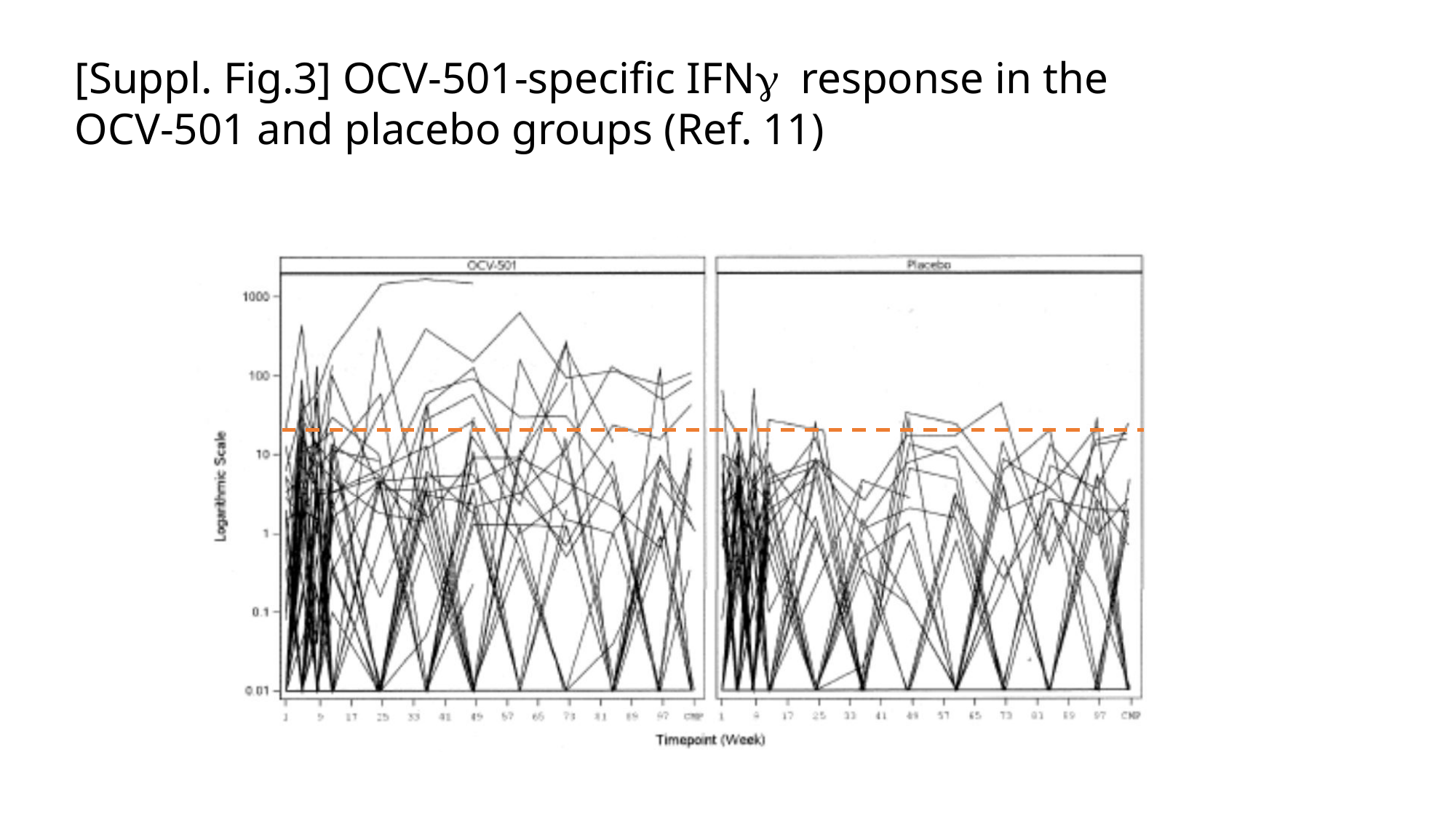

[Suppl. Fig.3] OCV-501-specific IFNg response in the OCV-501 and placebo groups (Ref. 11)
