# Supplementary material for: Immunoreactivity to WT1 peptide vaccine is associated with prognosis in elderly patients with acute myeloid leukemia: follow-up study of randomized phase II trial of OCV-501, an HLA class II-binding WT1 polypeptide
Source: Cancer Immunol Immunother. 2023 Apr 24;72(8):2865–71. doi: 10.1007/s00262-023-03432-4 (PMC10123586; doi:10.1007/s00262-023-03432-4)
Supplement: Supplementary file 2 — Supplementary file2 (DOCX 15 KB) [file 262_2023_3432_MOESM2_ESM.docx]

**Table 2 The anti-OCV-501 IgG level is an independent prognostic factor on DFS**

| Variables | | Univariate analysis | | |  | Multivariate analysis* | | |
| --- | --- | --- | --- | --- | --- | --- | --- | --- |
|  |  | HR | 95% CI | P-value |  | HR | 95% CI | P-value |
| WT1-specific IgG | low | 1 |  |  |  |  |  |  |
|  | intermediate | 0.58 | 0.26-0.1.30 | 0.18 |  |  |  |  |
|  | high | 0.21 | 0.08-0.58 | 0.002 |  | 0.21 | 0.08-0.58 | 0.002 |
| Age at diagnosis | per each additional year of age | 1.03 | 0.98-1.09 | 0.26 |  | - | - | - |
| Gender | Male | 1 |  |  |  |  |  |  |
|  | Female | 1.5 | 0.74-3.05 | 0.27 |  | - | - | - |
| ECOG PS | 0 | 1 |  |  |  |  |  |  |
|  | 1 or 2 | 1.35 | 0.74-2.43 | 0.33 |  | - | - | - |
| Disease risk | Myelodysplasia-related changes | 1 |  |  |  |  |  |  |
|  | Others | 0.68 | 0.26-1.80 | 0.44 |  | - | - | - |

The variables included in the multiple Cox regression analysis were WT1 specific IgG (low, intermediate vs. high), patient age at diagnosis, gender (male vs. female), ECOG PS (0 vs. 1-2), and disease risk (myelodysplasia-related changes vs. others). Any variables that were slightly associated with DFS in the univariate analyses (p<0.4) were considered for inclusion in the model. Significant variables associated with DFS were identified through stepwise selection. Reference groups: WT1 specific IgG low group, 60 years, male, PS=0, myelodysplasia-related changes.
